# Safety Monitoring of COVID-19 Vaccine Booster Doses Among Persons Aged 12–17 Years — United States, December 9, 2021–February 20, 2022

**DOI:** 10.15585/mmwr.mm7109e2

**Published:** 2022-03-04

**Authors:** Anne M. Hause, James Baggs, Paige Marquez, Winston E. Abara, Babatunde Olubajo, Tanya R. Myers, John R. Su, Deborah Thompson, Julianne Gee, Tom T. Shimabukuro, David K. Shay

**Affiliations:** ^1^CDC COVID-19 Emergency Response Team; ^2^Food and Drug Administration, Silver Spring, Maryland.

As of February 20, 2022, only BNT162b2 (Pfizer-BioNTech) COVID-19 vaccine has been authorized for use in persons aged 12–17 years in the United States (*1*). The Food and Drug Administration (FDA) amended the Emergency Use Authorization (EUA) for Pfizer-BioNTech vaccine on December 9, 2021, to authorize a homologous* booster dose for persons aged 16–17 years ≥6 months after receipt of dose 2 (*1*). On January 3, 2022, authorization was expanded to include persons aged 12–15 years, and for all persons aged ≥12 years, the interval between dose 2 and booster dose was shortened to ≥5 months (*1*). To characterize the safety of Pfizer-BioNTech booster doses among persons aged 12–17 years (adolescents), CDC reviewed adverse events and health impact assessments during the week after receipt of a homologous Pfizer-BioNTech booster dose reported to v-safe, a voluntary smartphone–based safety surveillance system for adverse events after COVID-19 vaccination, and adverse events reported to the Vaccine Adverse Event Reporting System (VAERS), a passive vaccine safety surveillance system managed by CDC and FDA. During December 9, 2021–February 20, 2022, approximately 2.8 million U.S. adolescents received a Pfizer-BioNTech booster dose.^†^ During this period, receipt of 3,418 Pfizer-BioNTech booster doses were reported to v-safe for adolescents. Reactions were reported to v-safe with equal or slightly higher frequency after receipt of a booster dose than after dose 2, were primarily mild to moderate in severity, and were most frequently reported the day after vaccination. VAERS received 914 reports of adverse events after Pfizer-BioNTech booster dose vaccination of adolescents; 837 (91.6%) were nonserious and 77 (8.4%) were serious. Health care providers, parents, and adolescents should be advised that local and systemic reactions are expected among adolescents after homologous Pfizer-BioNTech booster vaccination, and that serious adverse events are rare.

V-safe is a voluntary, smartphone–based U.S. active safety surveillance system established to monitor adverse events after COVID-19 vaccination (https://vsafe.cdc.gov/en/). The v-safe platform allows current registrants to report receipt of a booster dose of COVID-19 vaccine and new registrants to enter information about all doses received. Registrants aged ≤15 years must be enrolled by a parent or guardian. Health surveys are sent daily during the first week after administration of each dose and include questions about local injection site and systemic reactions and health impacts.^§^ CDC’s v-safe call center contacts registrants who indicate that medical care was sought after vaccination and encourages completion of a VAERS report, if indicated.

VAERS is a U.S. national passive vaccine safety surveillance system managed by CDC and FDA that monitors adverse events after vaccination (*2*). VAERS accepts reports from health care providers, vaccine manufacturers, and members of the public.^¶^ VAERS reports are classified as serious if there are any reports of hospitalization, prolongation of hospitalization, life-threatening illness, permanent disability, congenital anomaly or birth defect, or death.** VAERS staff members assign Medical Dictionary for Regulatory Activities (MedDRA) preferred terms to the signs, symptoms, and diagnostic findings in VAERS reports.^††^ Serious reports to VAERS were reviewed by CDC physicians to form a clinical impression based on available data. Reports of myocarditis and pericarditis, rare adverse events that have been associated with mRNA-based COVID-19 vaccines (*3*), after receipt of a booster vaccine were identified by a search for selected MedDRA preferred terms; CDC staff members attempted to collect information about clinical course and determined whether the CDC myocarditis case definition was met.^§§^

This report assessed local and systemic reactions and health impacts reported during the week after vaccination among adolescent v-safe registrants who received a homologous Pfizer-BioNTech booster dose ≥5 months after completion of their primary series during December 9, 2021–February 20, 2022. The odds of reporting an adverse reaction or health impact after dose 2 and booster dose were compared using a multivariable generalized estimating equations model; p<0.05 was defined as statistically significant.^¶¶^ VAERS reports for adolescents who received a Pfizer-BioNTech booster dose during December 9, 2021–February 20, 2022, were described by serious and nonserious classification, demographic characteristics (i.e., sex and age), and MedDRA preferred terms.*** Reporting rates for myocarditis were stratified by sex and age group. SAS software (version 9.4; SAS Institute) was used to conduct all analyses. These surveillance activities were reviewed by CDC and conducted consistent with applicable federal law and CDC policy.^†††^

## Review of v-safe Data

During December 9, 2021–February 20, 2022, v-safe recorded a total of 3,418 Pfizer-BioNTech booster doses administered to adolescents, including 1,952 administered to persons aged 12–15 years and 1,466 to those aged 16–17 years. Local injection site reactions (2,802; 82.0%) and systemic reactions (2,659; 77.8%) were frequently reported during the week after booster dose vaccination for all adolescents (Table 1); the most frequently reported adverse reactions were injection site pain (2,736; 80.0%), fatigue (1,998; 58.5%), headache (1,911; 55.9%), and myalgia (1,578; 46.2%). Reactions were mostly mild to moderate in severity and most frequently reported the day immediately after vaccination. Local injection site reactions were more commonly reported after booster dose (82.0%) than dose 2 (77.8%) (p<0.001), and systemic reactions were similarly reported after booster dose (77.8%) and dose 2 (77.2%) (p = 0.48) (Figure).

**TABLE 1:** Adverse reactions and health impacts reported to v-safe for persons aged 12–17 years* (N = 3,418) who received a homologous Pfizer-BioNTech COVID-19 vaccine booster dose — United States, December 9, 2021–February 20, 2022

Reported event	No. (%) reporting reaction or health impact after receipt of a homologous Pfizer-BioNTech vaccine^†^	
	Dose 2	Booster dose
**Any local injection site reaction**	**2,660 (77.8)**	**2,802 (82.0)**
Itching	250 (7.3)	252 (7.4)
Pain	2,596 (76.0)	2,736 (80.0)
Redness	287 (8.4)	350 (10.2)
Swelling	483 (14.1)	644 (18.8)
**Any systemic reaction**	**2,638 (77.2)**	**2,659 (77.8)**
Abdominal pain	318 (9.3)	291 (8.5)
Myalgia	1,399 (40.9)	1,578 (46.2)
Chills	949 (27.8)	1,115 (32.6)
Diarrhea	153 (4.5)	118 (3.5)
Fatigue	2,006 (58.7)	1,998 (58.5)
Fever	1,310 (38.3)	1,213 (35.5)
Headache	1,914 (56.0)	1,911 (55.9)
Joint pain	578 (16.9)	672 (19.7)
Nausea	643 (18.8)	647 (18.9)
Rash	52 (1.5)	41 (1.2)
Vomiting	93 (2.7)	78 (2.3)
**Any health impact**	**1,094 (32.0)**	**1,224 (35.8)**
Unable to perform normal daily activities	986 (28.8)	881 (25.8)
Unable to attend school or work	320 (9.4)	682 (20.0)
Needed medical care	21 (0.6)	32 (0.9)
Telehealth	4 (0.1)	6 (0.2)
Clinic	4 (0.1)	15 (0.4)
Emergency visit	8 (0.2)	5 (0.1)
Hospitalization	2 (0.1)	1 (0.03)

* Registrants aged ≤15 years must be enrolled by a parent or guardian.

^†^ Percentage of registrants who reported a reaction or health impact at least once during days 0–7 after vaccination.

**FIGURE:**
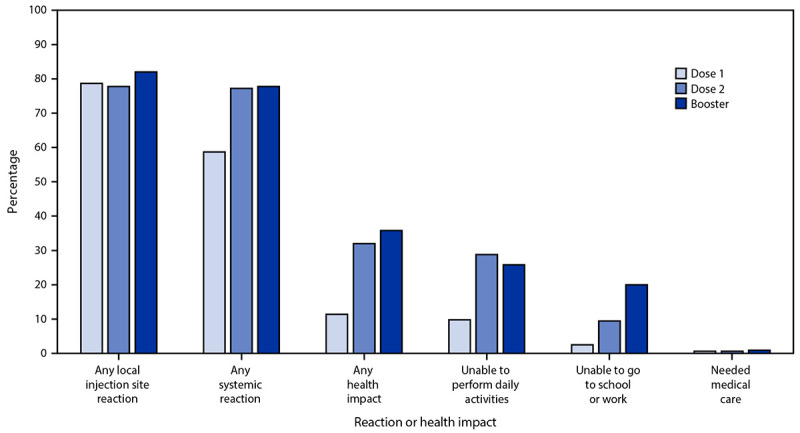
Adverse reactions and health impacts reported* among persons aged 12–17 years (N = 3,274) who received a homologous Pfizer-BioNTech COVID-19 vaccine booster, by vaccine dose — United States, December 9, 2021–February 20, 2022 * Registrants aged ≤15 years must be enrolled by a parent or guardian. The odds of reporting an event after dose 2 and booster dose were compared for registrants who completed at least one v-safe health check-in survey on days 0–7 after each vaccination using a multivariable generalized estimating equations model. This model adjusted for demographic variables and accounted for repeated measures among doses reported by each registrant (needed medical care was not adjusted due to small numbers); p <0.05 was considered statistically significant. All dose 2 and booster dose comparisons were statistically significant, except any systemic reaction and needed medical care.

In the week after booster dose vaccination, 20.0% (682) of adolescents were reported as being unable to attend school or work. Approximately 0.9% (32) of adolescents reportedly received medical care during the week after booster dose vaccination; most (15; 0.4%) care was received via a clinic appointment. One (0.03%) adolescent received care at a hospital during the week after booster dose vaccination for treatment of a new onset migraine; whether hospitalization was the result of vaccination could not be determined. Inability to perform daily activities was less frequently reported after receipt of the booster dose (25.8%) than after dose 2 (28.8%) (p<0.001) (Figure), whereas inability to work or attend school was more frequently reported (20.0% and 9.4%, respectively) (p<0.001). Receipt of medical care was more frequently reported after receipt of the booster dose than dose 2 (0.9% and 0.6%, respectively); however, the difference was not statistically significant (p = 0.12).

## Review of VAERS Data

During December 9, 2021–February 20, 2022, VAERS received and processed 914 reports of adverse events after receipt of a Pfizer-BioNTech booster dose for adolescents; the median age was 16 years, and 459 (50.2%) reports were for adolescent girls. Most VAERS reports were for nonserious events (837; 91.6%); the most commonly reported nonserious events included product storage error (123; 14.7%), dizziness (100; 12.0%), and syncope (87; 10.4%) (Table 2). Sixty-four preliminary reports of myocarditis were received, among which 47 were considered serious; 32 (68.1%) of these reports were confirmed by provider interview or medical record review to meet the CDC working definition of myocarditis. All 32 reports were among adolescent boys and 27 (84.4%) patients were hospitalized; as of February 20, 2022, all had been discharged, 18 had recovered, and nine were recovering. Among adolescent boys, the reporting rate for confirmed cases of myocarditis after Pfizer-BioNTech booster vaccination was 11.4 per 1 million booster doses administered. No deaths were reported to VAERS.

**TABLE 2:** Reports of nonserious and serious events to Vaccine Adverse Event Reporting System for persons aged 12–17 years (N = 914) who received a Pfizer-BioNTech COVID-19 vaccine booster — United States, December 9, 2021–February 20, 2022

Reported event	No. (%) reporting
**Nonserious VAERS reports**	
Symptom, sign, diagnostic result, or condition (MedDRA PT*)	**837 (100.0)**
Product storage error	123 (14.7)
Dizziness	100 (11.9)
Syncope	87 (12.0)
Fever	75 (9.0)
No adverse event^†^	70 (8.4)
Headache	69 (8.2)
Inappropriate schedule of product administration	56 (6.7)
Fatigue	55 (6.6)
Nausea	52 (6.2)
Pain	52 (6.2)
Expired product administered	40 (4.8)
Pain in extremity	40 (4.8)
Chest pain	39 (4.7)
Underdose	39 (4.7)
Vomiting	39 (4.7)
**Serious VAERS reports^§,¶^**	
**Clinical impression**	**77 (100.0)**
Myocarditis	47 (61.0)
Insufficient data to make a clinical impression	10 (13.0)
Appendicitis	3 (3.9)
Acute embolic stroke	2 (2.6)
Anaphylaxis or allergic reaction	2 (2.6)
Tachycardia	2 (2.6)
Acute pancreatitis	1 (1.3)
Exacerbation of existing genetic disorder	1 (1.3)
Guillain-Barré syndrome	1 (1.3)
Immune thrombocytopenia	1 (1.3)
Injection site pain	1 (1.3)
Pericardial effusion	1 (1.3)
Rhabdomyolysis	1 (1.3)
Severe headache	1 (1.3)
Side effect of prescription medication	1 (1.3)
Spontaneous tension pneumothorax	1 (1.3)
Transverse myelitis	1 (1.3)

**Abbreviations:** MedDRA = Medical Dictionary for Regulatory Activities; PT = preferred term; VAERS = Vaccine Adverse Event Reporting System.

* Signs and symptoms in VAERS reports are assigned MedDRA PTs by VAERS staff members. Each VAERS report might be assigned more than one MedDRA PT, which can include normal diagnostic findings; thus, the events listed in the table might sum to more than the total number of reports. A MedDRA PT does not indicate a medically confirmed diagnosis.

^†^ Reports of no adverse event were often accompanied by product storage error, inappropriate schedule of product administration, expired product administered, or underdose.

^§^ VAERS reports are classified as serious if there are any reports of hospitalization, prolongation of hospitalization, life-threatening illness, permanent disability, congenital anomaly or birth defect, or death.

^¶^ Serious reports to VAERS were reviewed by CDC physicians to form a clinical impression. Reports of myocarditis were identified using a combination of MedDRA PTs; in some cases, reports of myocarditis (identified by fulfilling criteria of the CDC working case definition of myocarditis) did not have the MedDRA PT “myocarditis” assigned to them. https://www.meddra.org/how-to-use/basics/hierarchy

## Discussion

This report provides findings from v-safe and VAERS data collected during the first 7–11 weeks of administration of homologous Pfizer-BioNTech booster doses to persons aged 12–17 years, during which time approximately 2.8 million booster doses were administered. Among adolescents, reports to v-safe and VAERS after receipt of a booster dose were generally similar to those previously described after a primary series dose, reinforcing that vaccination among this population is safe (*4*,*5*). Health care providers, parents, and adolescents should be advised that local and systemic reactions are expected among adolescents after Pfizer-BioNTech booster vaccination and that serious adverse events are rare.

Reports to v-safe after receipt of a booster dose in an adolescent were generally similar to those previously described for persons aged ≥18 years who received a homologous booster dose of Pfizer-BioNTech vaccine (*6*,*7*); however, reactions among adolescents were reported to v-safe with equal or slightly higher frequency after receipt of a booster dose than after dose 2. Reactions reported after both dose 2 and booster dose vaccination were mostly mild to moderate in severity. Most were reported the day after vaccination. Inability to attend school was more frequently reported after a booster dose than after dose 2; however, for many in this age group, receipt of dose 2 occurred during a period of remote learning or summer vacation, which might have affected reporting. Hospitalization in the week after booster dose vaccination was reported for one adolescent with new onset migraine; whether hospitalization was the result of COVID-19 vaccination could not be determined.

Most (91.6%) reports to VAERS for adolescents after a Pfizer-BioNTech booster dose were nonserious and generally similar to those reported for this age group after primary series vaccination (*4*). The most common adverse events reported to VAERS in this age group were administration errors and events, including dizziness, related to syncope, a vasovagal response to vaccination that is common among adolescents after any vaccination (*8*). Most reports of administration errors mentioned that no adverse event was associated with receipt of an incorrect dose.

Among the 64 VAERS reports of myocarditis, a rare adverse event that has been associated with mRNA-based COVID-19 vaccines (*3*), after Pfizer-BioNTech booster dose vaccination among adolescents, 32 cases were confirmed at the time of this report. The reporting rate of confirmed cases of myocarditis among adolescent boys after Pfizer-BioNTech booster dose vaccination (11.4 per 1 million doses administered) was lower than for dose 2 Pfizer-BioNTech vaccination for boys aged 12–15 years (70.7 per 1 million doses administered) or 16–17 years (105.9 per 1 million doses administered) (*3*). CDC will follow up on myocarditis reports at 3–6 months after onset to assess health and functional status.

The findings in this report are subject to at least four limitations. First, v-safe is a voluntary program; therefore, data might not be representative of the vaccinated population. Second, it is possible that vaccinees who experience an adverse event could be more likely to respond to v-safe surveys. Third, as a passive surveillance system, VAERS is subject to reporting biases and underreporting, especially of nonserious events (*2*). Finally, assessment of myocarditis reports to VAERS is ongoing, and counts are subject to change.

The Advisory Committee on Immunization Practices recommends that all persons aged ≥12 years receive a booster dose of COVID-19 vaccine ≥5 months after the second dose of the mRNA vaccine primary series (*9*). Preliminary safety findings for booster doses among adolescents are generally similar to those reported after a primary series in this age group. Health care providers, parents, and adolescents should be advised that local and systemic reactions are expected among adolescents after homologous Pfizer-BioNTech booster vaccination, and that serious adverse events are rare. CDC and FDA will continue to monitor vaccine safety and will provide updates as needed to guide COVID-19 vaccination recommendations.

**Summary**

**What is already known about this topic?**

Adults aged ≥18 years reported adverse reactions less frequently after receipt of a homologous Pfizer-BioNTech COVID-19 booster dose than after the second primary dose.

**What is added by this report?**

Among persons aged 12–17 years, reactions after Pfizer-BioNTech booster vaccination were generally mild to moderate and transient; the frequency of local and systemic reactions reported to v-safe after a booster dose were equal to or slightly higher than after the second primary dose. Myocarditis was less frequently reported after a booster dose than a second primary dose.

**What are the implications for public health practice?**

Health care providers, parents, and adolescents should be advised that local and systemic reactions are expected among adolescents after a homologous Pfizer-BioNTech booster vaccination and that serious adverse events are rare.

## Footnotes

* Homologous refers to a booster dose of the same product administered for the primary series.

†
https://covid.cdc.gov/covid-data-tracker/#vaccination-demographic

§ Health surveys are sent for the most recent dose entered via text messages that link to web-based surveys on days 0–7 after vaccination; then weekly through 6 weeks after vaccination; and then 3, 6, and 12 months after vaccination.

¶ Health care providers are required by COVID-19 vaccine EUAs to report certain adverse events after vaccination to VAERS, including death. https://vaers.hhs.gov/faq.html

** VAERS reports are classified as serious based on the Code of Federal Regulations Title 21 (https://www.accessdata.fda.gov/scripts/cdrh/cfdocs/cfcfr/cfrsearch.cfm?fr). Reports of serious adverse events receive follow-up by VAERS staff to obtain additional information, including medical records and, for reports of death, death certificates and autopsy reports, if available.

†† Each VAERS report might be assigned more than one MedDRA preferred term. A MedDRA-coded event does not indicate a medically confirmed diagnosis. https://www.meddra.org/how-to-use/basics/hierarchy

§§ Acute myocarditis was defined as presence of signs and symptoms (one or more new or worsening of the following: chest pain/pressure/discomfort, dyspnea/shortness of breath/pain with breathing, palpitations, or syncope; or two or more of the following in children aged ≤11 years: irritability, vomiting, poor feeding, tachypnea, or lethargy); and one or more new finding of elevated troponin, electrocardiogram findings consistent with myocarditis, abnormal cardiac function or wall motion on echocardiogram, cardiac magnetic resonance imaging findings consistent with myocarditis, or histopathologic findings consistent with myocarditis; and no other identifiable cause for these findings.

¶¶ This model adjusted for demographic variables (i.e., age, sex, race, and ethnicity) and accounted for repeated measures among doses reported by each registrant.

*** This analysis excluded reports to v-safe or VAERS of persons aged 12–15 and 16–17 years who were vaccinated before authorization for a booster dose for their age group (January 3, 2022, and December 9, 2021, respectively).

††† 45 C.F.R. part 46, 21 C.F.R. part 56; 42 U.S.C. Sect. 241(d); 5 U.S.C. Sect. 552a; 44 U.S.C. Sect. 3501 et seq.
